# Effect of the Degree of Conversion on Mechanical Properties and Monomer Elution from Self-, Dual- and Light-Cured Core Composites

**DOI:** 10.3390/ma14195642

**Published:** 2021-09-28

**Authors:** Branislava Petronijevic Sarcev, Sebastian Balos, Dubravka Markovic, Ivan Sarcev, Marija Vukcevic, Danka Labus Zlatanovic, Vesna Miletic

**Affiliations:** 1 Medical Faculty, University of Novi Sad, Hajduk Veljkova 3, 21000 Novi Sad, Serbia; branislava.petronijevic@mf.uns.ac.rs (B.P.S.); dubravka.markovic@mf.uns.ac.rs (D.M.); ivan.sarcev@mf.uns.ac.rs (I.S.); 2 Faculty of Technical Sciences, University of Novi Sad, Trg Dositeja Obradovica 6, 21000 Novi Sad, Serbia; sebab@uns.ac.rs (S.B.); or danka.labus-zlatanovic@tu-ilmenau.de (D.L.Z.); 3 Faculty of Technology and Metallurgy, University of Belgrade, Karnegijeva 4, 11000 Belgrade, Serbia; marijab@tmf.bg.ac.rs; 4 Department of Production Technology, Technische Universität Ilmenau, 98693 Ilmenau, Germany; 5 Faculty of Medicine and Health, Sydney Dental School, The University of Sydney, 2 Chalmers Street, Surry Hills 2010, Australia

**Keywords:** composite core materials, mechanical properties, degree of conversion, monomer elution

## Abstract

The objective of this work was to measure and correlate the degree of conversion (DC), mechanical properties and monomer elution from self-, dual- and light-cured core composites. Five samples of each of the following materials were prepared for each test: Clearfil (Core, Photo Core, Automix), Bisco (Core-Flo, Light-Core and Bis-Core). DC was determined using FTIR, compressive and flexural strength and modulus of elasticity using a universal testing machine and microhardness using Vickers hardness. Elution was measured using HPLC. One-way ANOVA with Tukey’s post-test and Pearson’s correlation were used to statistically analyze the data. DC of Clearfil-Dual (70.1%) and Clerafil-Photo (66.8%) were higher than Clearfil-Self (55.4%) and all Bisco materials (51.4–55.3%). Flexural strength of Clearfilwas higher than that of Bisco composites. The Microhardness of Clearfil-Dual (119.8VHN) and Clearfil-Photo (118.0VHN) were higher compared to other materials. The greatest elution was detected from self-cured materials. DC positively correlated to microhardness and compressive/flexural strength and negatively to BisGMA elution. Clearfil-Photo and Automix showed higher conversion, lower monomer elution and, generally, better mechanical properties. Self-cured composites should not be recommended for routine clinical use as their performance was inferior to dual- and light-cured composites. Microhardness may be used as an indicator of elution.

## 1. Introduction

Core build-up composites (core composites) are specifically designed to restore the coronal portion of a severely destroyed tooth prior to the placement of an indirect restoration. These materials are stated by the manufacturers to have high strength, hardness and increased depth of cure providing reliable support for the overlying restoration.

Light-cured universal composites for direct restorations have undergone substantial improvements over the years [1,2] becoming an established standard of care [3]. The reported annual failure rates of up to 3% [4,5] suggest that light-cured composites are reliable even when placed by inexperienced operators [5,6]. On the other hand, self- and dual-cured core composites are recommended for core build-ups especially in areas with unfavourable C-factors and/or areas inaccessible for light curing. The slow setting of self-cured composites is considered beneficial for stress relief in areas such as the entrance of the root canal or parts of the pulp chamber with opposing cavity walls [7]. 

Several studies compared the mechanical properties of core composites with other materials previously used for the same purpose, concluding that all materials complied with the minimum requirements [8,9,10,11]. Higher shrinkage and lower expansion in silicone oil and distilled water were reported for self- and dual-cured core composites compared to a metal-reinforced and a resin-modified glass-ionomer cement [12]. Self-cured Clearfil Core has shown lower contraction stress than that of the light-cured counterpart Clearfil Photo Core in cavities with C-factor = 3 [7]. C factor is defined as the ratio of the bonded to the unbonded surface area and depends on the cavity configuration [13]. Polymerization shrinkage is well correlated with shrinkage stress in four dual-cured core composites [14]. Another study reported that the hardness decreases in dual-cured Bisco core composite as the depth of the root canal increases due to a longer distance and a lower effect of the polymerization light [15].

Bond strength to dentine of core composites has been affected to a greater extent by polymerization of the accompanying adhesive than that of the core material itself [16]. In another study, four dual-cured core composites showed comparable microtensile bond strength to root canal dentine despite the differences in ultimate tensile strength and Knoop hardness [17]. Light-curing in dual-cured core composites was reported to increase the degree of conversion (DC) and bond strength to dentine compared to chemical-curing alone but the curing regime, standard or exponential, did not affect these properties [18]. Sadek et al. [19] showed comparable or higher bond strength to fiber posts of the core compared to hybrid and flowable composites.

Elution studies have found both organic and inorganic substances leaching out of self- and dual-cured core composites in various extraction media [20,21,22,23]. Core composites have shown in vitro cytotoxicity in direct contact with vital cells [24,25] which may be attributed largely to the toxicity of monomers [23,26,27,28].

There are no literature data on the relationship between the quality of polymerization of core composites and their mechanical properties and elution of potentially harmful substances. Therefore, this study aimed to compare the DC, mechanical properties and monomer elution from self-, dual- and light-cured core composites from the same manufacturer and study the correlation between these properties. The null hypotheses were: (1) there are no differences in the tested properties between composites with different types of polymerization, and (2) there is no correlation between the DC, mechanical properties and elution for the tested core composites.

## 2. Materials and Methods

Six core build-up composite materials were tested, with their description data given in Table 1. For flexural strength and modulus of elasticity measurements, 2 × 2 × 25 mm samples were prepared as specified in the standard ISO 4049 [26]. For compressive strength testing, microhardness, DC and monomer elution measurements, cylindrical specimens 5 mm in diameter and 2 mm thick were prepared using standardized moulds.

Five samples from the group per test were prepared. Self- and dual-cured materials were mixed according to the manufacturer’s instructions. A mould was placed on a glass plate, filled with material, pressed with a Mylar strip to extrude excess and prevent oxygen inhibition layer. Each material was polymerized according to the manufacturer’s instructions. Clearfil-Self and Bisco-Self were allowed to chemically cure for 5 min. Clearfil-Photo and Bisco-Photo were cured for 20 s whilst Clearfil-Dual and Bisco-Dual were cured for 10 s using an LED light-curing unit (Bluephase, IvoclarVivadent, Schaan, Liechtenstein), operating at an intensity of 1200 mW/cm^2^. The tip-to-surface distance was standardized to 1 mm and was maintained with a custom-made guide. Immediately after light curing, excess material was removed with a scalpel and each sample was polished using a series of Sof-Lex discs (medium, soft, super-soft, 3M ESPE, St. Paul, MN, USA) for 30 s each, under water and standardized pressure. The samples were then stored in dark light-proof containers at 37 °C for 24 h, except for monomer elution where samples were immersed 10 min post-curing.

### 2.1. Degree of Conversion

The DC was evaluated using an FTIR spectrometer (Thermo Nicolet Nexus 670 FTIR spectrometer, Medison, WI, USA) under the following conditions: 32 scans across the 4000-400 cm^−1^ range at a resolution of 4 cm^−1^.

The monomer-to-polymer conversion was determined using a two-frequency technique based on an analytical and a reference frequency. The DC was calculated using Equation (1):

(1) $$DC\%=\left(1-\frac{R_{cured}}{R_{uncured}}\right)\cdot 100$$

where *R* is the ratio of the absorbance peak at 1638 cm^−1^ corresponding to the aliphatic groups and absorbance peak at 1608 cm^−1^ corresponding to the aromatic groups in the spectra of cured or uncured samples. Spectra of the unset pastes obtained during the same session were used as controls.

### 2.2. Flexural Strength, Modulus of Elasticity, Compressive Strength and Microhardness

To determine flexural strength and modulus of elasticity, a three-point bending test was conducted using a universal testing machine (Toyoseiki AT-L-118B, Tokyo, Japan) at a crosshead speed of 1 mm/min. Flexural strength was calculated using Equation (2):

(2) $$\sigma =\frac{3\cdot F_{max}\cdot L}{2\cdot B\cdot H^{2}}$$

where *σ* is flexural strength [MPa], *F_max_* is maximum load [N], *L* is the distance between supports [mm], *B* is the width of the specimen [mm] and *H* is the height of the specimen [mm].

Modulus of elasticity was calculated using Equation (3):

(3) $$E=\frac{F\cdot L^{3}}{4\cdot B\cdot H^{3}\cdot d}$$

where *E* is the modulus of elasticity [GPa], d is the deflection [mm] that corresponds to the load *F* and *F*, *L*, *B* and *H* are as in the previous equation.

Compressive strength was tested using a universal testing machine (Model 282, VEB, Leipzig, Germany). Strength values were calculated according to Equation (4):

(4) $$C=\frac{4\cdot F_{max}}{\pi \cdot d^{2}}$$

where *C* is compressive strength [MPa], *F_max_* is maximum load [N] and *d* is specimen diameter [mm].

Microhardness was measured using a Vickers microhardness tester (HVS-1000; Huayin, Laizhou, China) and the following parameters: 100 g load and 15 s dwell time. For every sample, five indentations were carried out and microhardness was determined as an average value of calculated Vickers hardness numbers (VHN) by using Equation (5):

(5) $$VHN=1.8544\cdot \frac{F}{d^{2}}$$

where *VHN* is the Vickers hardness number [VHN], *F* is the load [kg] and *d* is the average between indentation diagonals [mm].

### 2.3. Monomer Elution

Each sample was immersed in 1mL of 75% ethanol/water solution (HPLC Gradient Grade solvents) in a glass, dark vial and stored at 37 °C. HPLC measurements were done 28 days post-immersion. The whole solution was taken for analysis.

Qualitative and quantitative analysis was performed on an HPLC instrument (Thermo Fisher Scientific Inc., Waltham, MA, USA) equipped with an XDB-C18 column, 75 mm long, 4.6 mm inner diameter and 3.5 μm particle size (Zorbax Eclipse^®^, Agilent Technologies, Santa Clara, CA, USA). In front of the separation column, a pre-column was installed, 12.5 mm long, 4.6 mm inner diameter and 5 μm particle size (Zorbax Eclipse^®^, Agilent Technologies, Santa Clara, CA, USA). The mobile phase was a mixture of water and acetonitrile (HPLC Grade, Sigma–Aldrich, Dorset, UK) and a gradient was applied according to the following method (A: H_2_O, B: CH_3_CN): 40% B (0–4 min); 70% B (4–9 min); 100% B (9–11 min); 40% B (11.01–17 min). The flow rate was 1 mL/min and the injection volume was 10 μL. UV detection was performed at 205 nm (for monitoring the elution of BisGMA and TEGDMA) and 275 nm (for monitoring the elution of BisGMA). The compounds were identified by comparison of their retention times with those of the reference compounds under the same HPLC conditions. The retention times of the reference standards were 4.44 min (TEGDMA) and 6.67 min (BisGMA).

Reference standards of BisGMA and TEGDMA (>98% purity; IvoclarVivadent, Schaan, Liechtenstein) were used to produce stock solutions of 1000 μg/mL each. The stock solutions were diluted in acetonitrile to produce the final calibration solutions 2.5, 5, 10, 15 and 20 μg/mL. The peak area for each monomer was plotted versus concentration using linear regression analysis to quantify monomer concentration in the sample solutions. The limits of quantification (LoQ) and detection (LoD) were calculated for a signal-to-noise ratio of 10 (S/N = 10) and 3 (S/N = 3), respectively. The LoQ was 0.325 μg/mL for TEGDMA and 0.442 μg/mL for BisGMA. The LoD was 0.097 μg/mL for TEGDMA and 0.132 μg/mL for BisGMA.

### 2.4. Statistical Analysis

Statistical analysis was performed in Minitab 16 (Minitab Inc, State College, PA, USA). The data were analyzed using a one-way analysis of variance (ANOVA) with Tukey’s post-hoc test. Correlation between the DC, mechanical properties and elution of monomers was analyzed using Pearson’s correlation. The significance level was set at α = 0.05.

## 3. Results

**Figure 1:**
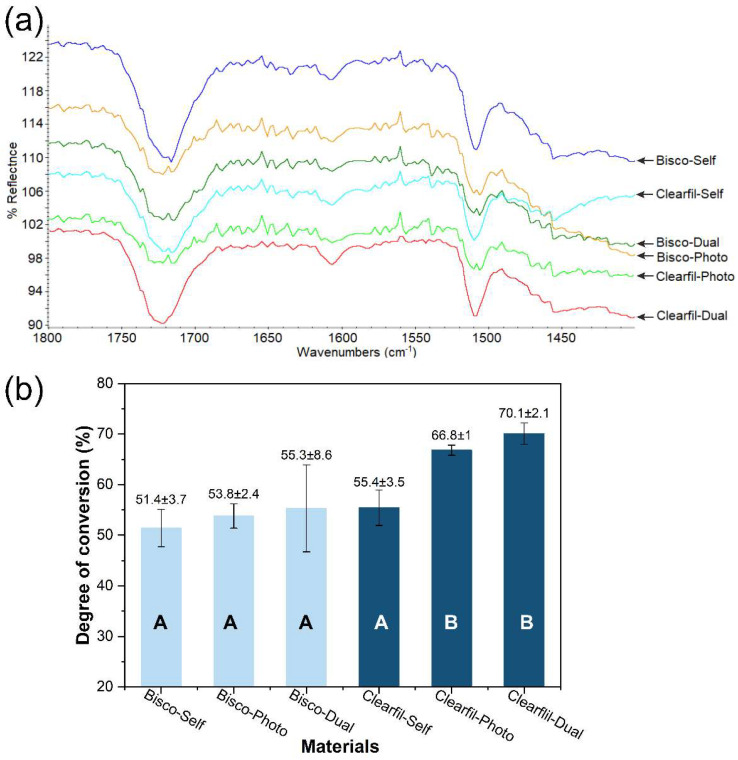
Mean and standard deviation values of the degree of conversion of core composites. The same letters indicate no significant difference between materials (*p* > 0.05): (**a**) FTIR spectra and (**b**) degree of conversion of tested materials.

DC of tested materials, along with corresponding FTIR spectra are shown in Figure 1. It can be seen that Clearfil-Dual and Clerafil-Photo showed significantly higher DC than Clearfil-Self and all three Bisco materials (*p* < 0.05). There were no significant differences between Bisco materials irrespective of the type of polymerization (*p* > 0.05), although a slightly higher mean was found for Bisco-Dual.

**Figure 2:**
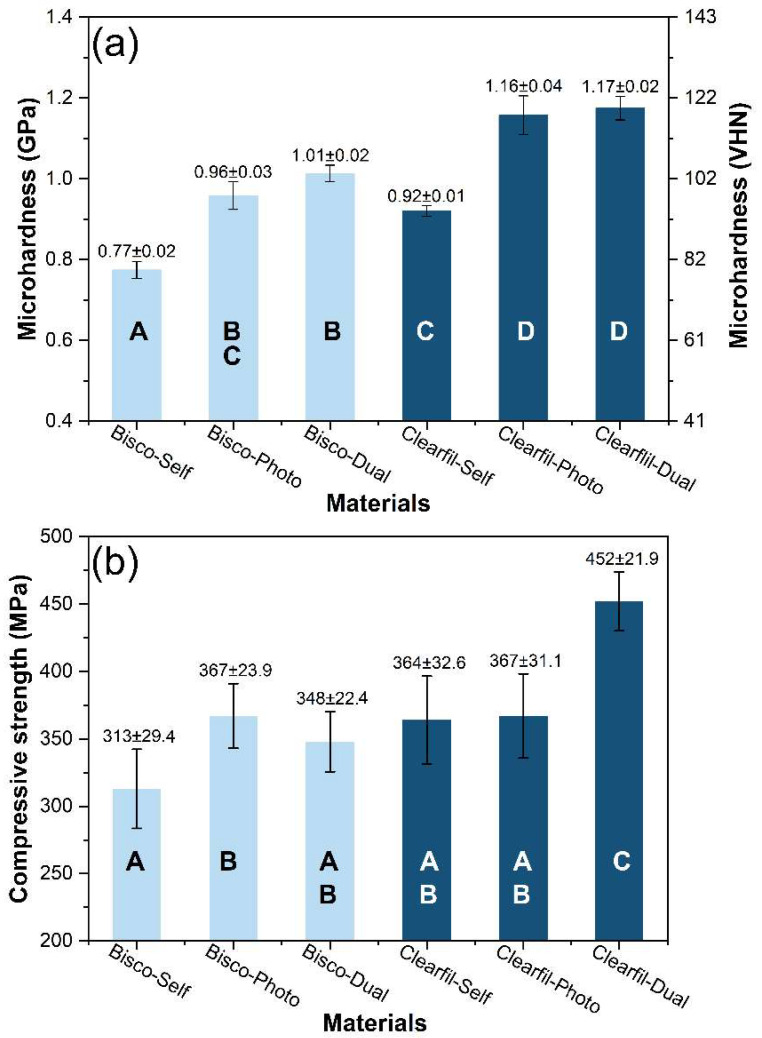
Mean and standard deviation values of (**a**) microhardness and (**b**) compressive strength of core composites. The same letters indicate no significant difference between materials (*p* > 0.05).

**Figure 3:**
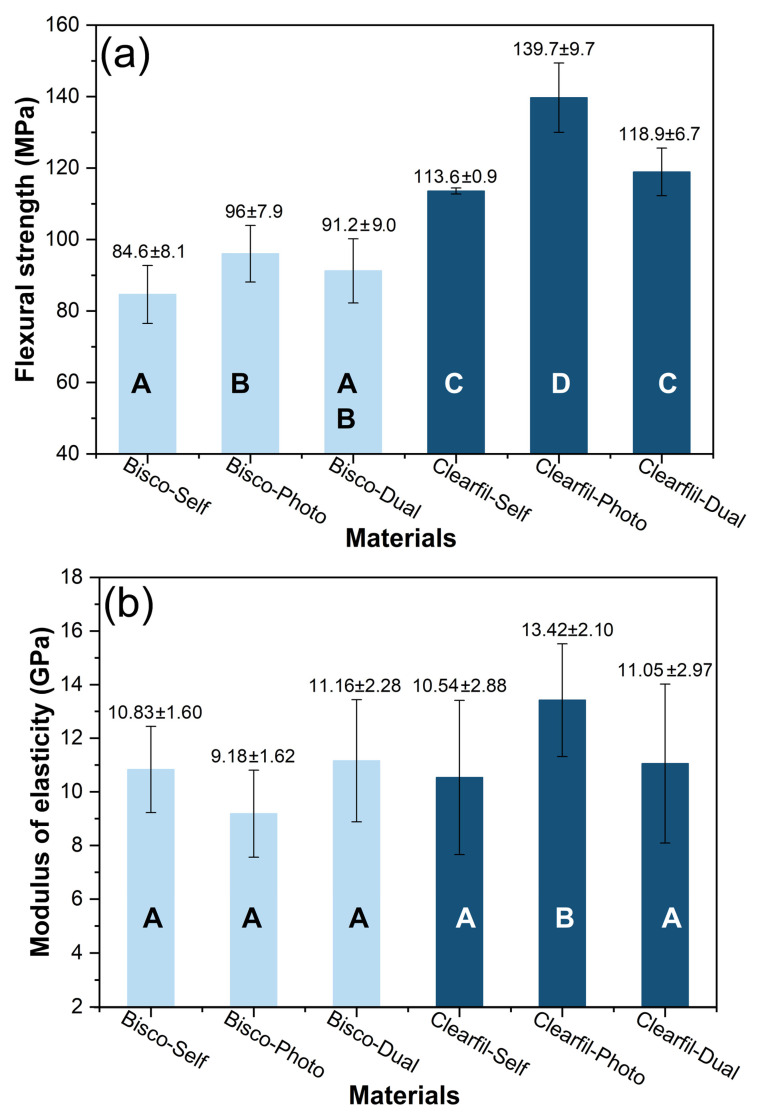
Mean and standard deviation values of (**a**) flexural strength and (**b**) modulus of elasticity of core composites. The same letters indicate no significant difference between materials (*p* > 0.05).

Figure 2 and Figure 3 present the data of mechanical properties of the tested core composites. The Microhardness of Clearfil-Dual (119.8 VHN) and Clearfil-Photo (118.0 VHN) were significantly higher compared to other materials (*p* < 0.05). Clearfil-Dual showed significantly higher compressive strength (452 MPa) than all other tested materials (*p* < 0.05) which were in the range of 310–370 MPa. All three Clearfil composites showed higher flexural strength than Bisco composites (*p* < 0.05) whereas the differences in modulus of elasticity were not so pronounced. Mechanical properties of self-cured core composites were similar or inferior to dual- and light-cured materials.

Table 2 illustrates the differences in monomer content claimed by the manufacturers and the actual measured amounts using HPLC. The greatest mismatch was found for the Bisco-Self base and catalyst where the measured amounts were 3–4 times smaller than the maximum values claimed by the manufacturer.

**Figure 4:**
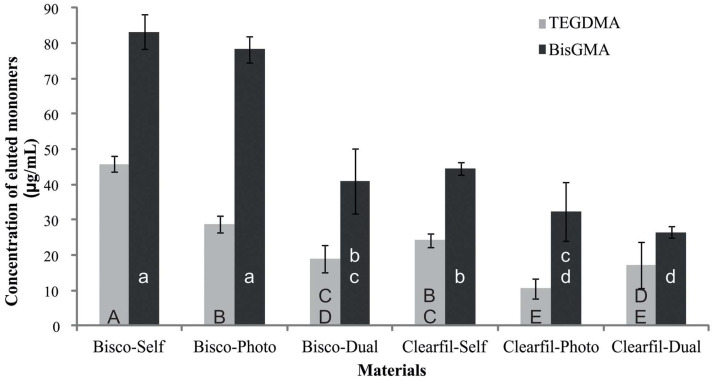
Mean and standard deviation values of monomer elution from core materials. The same upper-case letters indicate no significant differences in TEGDMA elution; the same lower case letters indicate no significant differences in BisGMA elution (*p* > 0.05).

In all groups, elution of BisGMA, ranging between 26 and 83 µg/mL, was greater than that of TEGDMA (10–46 µg/mL). Elution was greater from Bisco-Self and Clearfil-Self compared to their light- and dual-cured counterparts (*p* < 0.05). In general, greater elution was found from Bisco than Clearfil materials (Figure 4). 

**Figure 5:**
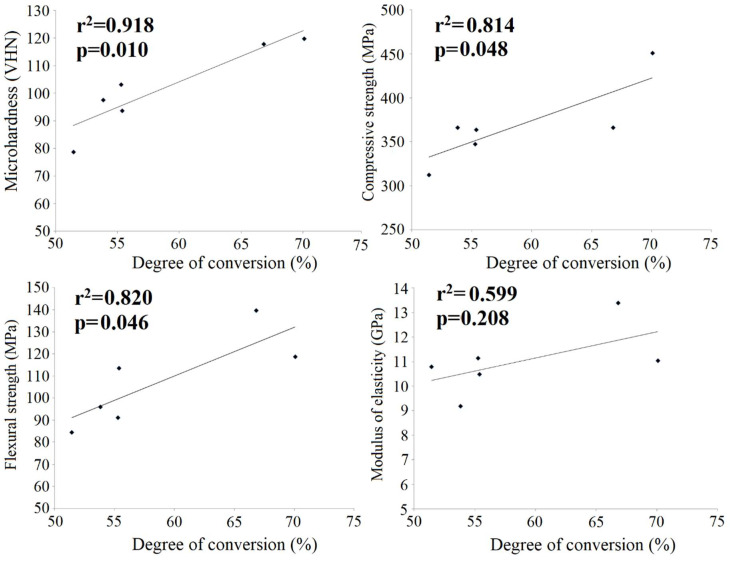
Correlation between mechanical properties and the degree of conversion of core composites.

**Figure 6:**
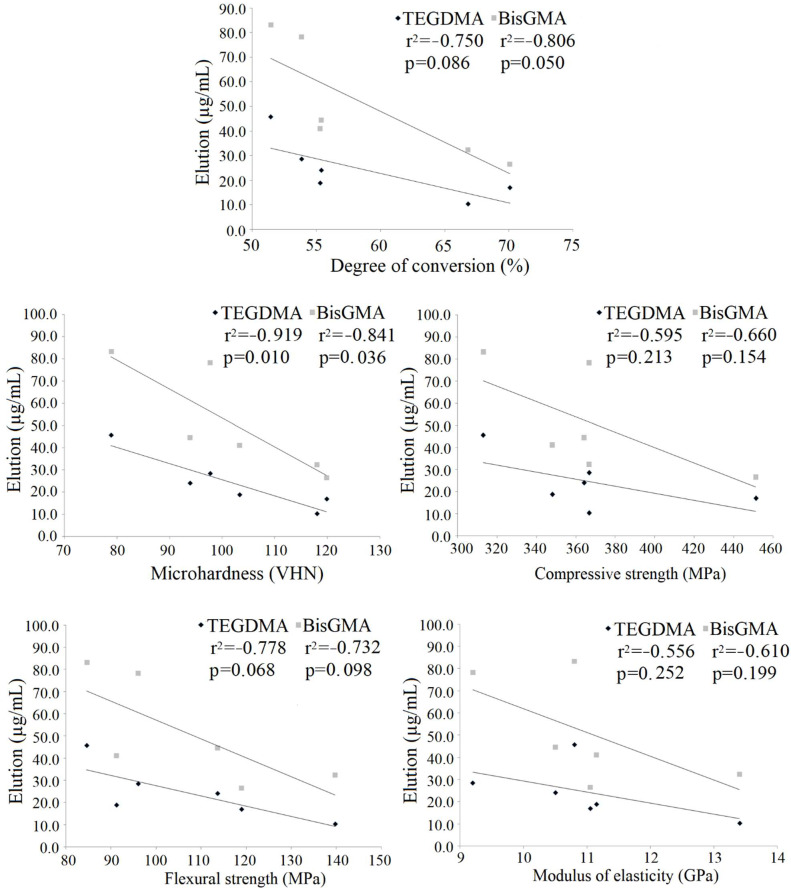
Correlation between monomer elution and the degree of conversion and between monomer elution and mechanical properties of core composites.

Pearson’s correlations are illustrated in Figure 5 and Figure 6 with the Pearson correlation coefficients (r2) and p values indicating statistical significance. A strong positive correlation was found for the DC and microhardness whilst a weak correlation existed between the DC and compressive/flexural strength. Conversely, a strong negative correlation was found between microhardness and TEGDMA elution whereas weak negative correlations existed between BisGMA elution and the DC/microhardness.

## 4. Discussion

Both null hypotheses were rejected as significant differences were found in the DC, mechanical properties and monomer elution between the core composites. Materials chosen for this study differed in the type of polymerization, being either self-, light- or dual-cured. Composites from the same manufacturer were chosen as manufacturers tend to have the same underlying philosophy regarding material composition. All tested composites were based on BisGMA and TEGDMA cross-linking monomers with the addition of small amounts of other monomers. On the other hand, filler content varied considerably in the range of 52–75 wt%. Such material selection allowed at least some variables to be controlled although this is exceedingly difficult in commercial materials.

Mechanical properties of core materials theoretically depend on a number of factors related to the polymer matrix, as well as filler particles, similarly to universal composites [29,30,31,32]. The present results indicate that the type of curing reaction affects monomer-to-polymer conversion, material properties, as well as elution of monomers. Not only does the polymer itself possess certain mechanical characteristics, but also unreacted monomers may act as void, creating crack initiation sites that may weaken the material during stresses.

Generally, Clearfil-Photo and Clearfil-Dual showed better mechanical properties than other materials. This finding is in agreement with the DC values, which were the highest in these two groups. The lowest overall mechanical properties were found for Bisco-Self, also having the lowest DC, despite high filler content (up to 75 wt%). Based on the present results, light- and dual-cured core materials may be considered superior to self-cured materials. There are no studies in the literature with a similar methodology to compare the present data to.

A previous study found lower hardness values for Clearfil-Dual core composite following placement in post space in root canals [17]. In another study, despite differences in the DC, chemically- and light-cured Clearfil core composites showed similar bond strength to dentine with adhesives having a greater effect on bond strength than composites [7].

In this study, Clearfil-Dual had similar microhardness and yet significantly higher compressive strength compared to Clearfil-Photo. This difference may come from the testing concept. During compression testing, the whole specimen volume is loaded, which may mimic the differences in regional structural properties. Conversely, microhardness represents the resistance of a material to local plastic (permanent) deformation exerted by a harder indenter.

The three-point bending test has shown higher flexural strength and modulus of elasticity of Clearfil-Photo than Clearfil-Dual. Since polymerization of these two materials was of similar quality, fillers may have affected flexural strength and modulus of elasticity. Localize stress during the three-point bending test may promote the impact of the filler content. In composites, the maximum load under the ram is transferred from the matrix to the filler particles and theoretically, flexural strength and modulus of elasticity fall within two bounds expressed through Equations (6) and (7) [33]:

(6) $$E_{c}=f_{r}\cdot E_{r}+\left(1-f_{r}\right)\cdot E_{m}$$

(7) $$\frac{1}{E_{c}}=\frac{f_{r}}{E_{r}}+\frac{\left(1-f_{r}\right)}{E_{m}}$$

where *E_c_* is the modulus of elasticity of the composite material [GPa], *f_r_* is the reinforcing particles (filler), *E_r_* is the modulus of elasticity of the filler [GPa] and *E_m_* is the modulus of elasticity of the matrix [MPa].

As can be seen from Equations (6) and (7), both matrix and reinforcing particles (filler) contribute to the strength and modulus of elasticity of composites. When the effect of the organic matrix is similar, as seen here for Clearfil-Photo and Clearfil-Dual, the impact of filler particles may be decisive. HPLC analysis confirmed lower monomer and, thus, higher filler content in Clearfil-Photo compared to Clearfil-Dual, explaining its higher flexural strength and modulus of elasticity.

Bisco-Photo and Bisco-Dual have shown similar DC and mechanical properties although monomer elution was higher from Bisco-Photo. These differences may be due to the differences in the structure of the polymer network, which may (1) facilitate monomer elution through swelling or (2) differ in the amount of unreacted monomers even when the DC values are comparable. In a previous study, the microhardness of a light-cured Bisco core composite applied to the root canal did not exceed 90 VHN which was lower compared to the present values of around 100 VHN [15]. Longer curing times were found to increase the hardness of light-cured Bisco core [15] whereas pre-heating this composite did not affect surface hardness [34].

BisGMA eluted more than TEGDMA from all tested materials which is in agreement with previous studies on Clearfil core composites [22,35]. This finding is most likely due to higher BisGMA content which was confirmed by HPLC analysis of uncured materials. TEGDMA is often added to dilute highly viscous BisGMA albeit in small amounts for an optimal balance between curing reactivity and mechanical properties of the polymer [30]. The actual measured amounts of eluted BisGMA and TEGDMA from Clearfil materials differed from the study by Polydorou et al. [22] as much as 9-10 times for Clearfil-Self and 4 times for Clearfil-Dual. The smallest differences between the two studies were found for Clearfil-Photo, i.e., BisGMA elution was double that of the present result whereas TEGDMA elution was comparable with the present study. Since the parameters of the present and previous study [22] were fairly similar, such large differences in monomer elution from Clearfil-Self and Clearfil-Dual should be attributed to variations in sample preparation, i.e., dosage and mixing of base and catalyst. As expected, these variations were more pronounced in the case of the self-cured than dual-cured material, which is in agreement with the study of Karakis et al. [36]

A strong positive correlation existed between the DC and microhardness and a weak correlation between the DC and compressive and flexural strength. This may be explained to some extent by the fact that both DC and microhardness are measured on a point-sized area whereas compressive and flexural strengths are measured over the entire sample. Similarly, previous studies have shown that material properties correlate with curing parameters that affect monomer conversion [37] or the DC itself [38].

Monomer elution was inversely proportional to the DC and microhardness, but no relationship was found between elution and compressive, flexural strength and modulus of elasticity. Previous studies correlated other mechanical properties of core composites and found no correlation between the ultimate tensile strength and hardness [17], whereas linear polymerization shrinkage correlated well with shrinkage stress [14]. The present results suggest that microhardness may be used as an indicator of monomer elution. Furthermore, monomer elution may be used not only as an indicator of biocompatibility but also of the quality of polymerization of core composites.

It was interesting to notice that some manufacturers’ claims about the amount of monomer content varied significantly from the measured amounts. This indicates that relying solely on manufacturers’ data may be misleading and doubts the stated amounts of other ingredients. Since it is impractical for researchers to verify the chemical composition of every tested material, manufacturers are urged to provide more accurate data.

## 5. Conclusions

In accordance with the presented results in this study, the following conclusions can be drawn:

- Clearfil-Photo and Clearfil-Dual showed higher conversion, lower monomer elution and, generally, better mechanical properties than Clearfil-Self and Bisco core composites. This indicates that light and combined light and self-curing are more effective curing methods, which is of significance for clinical practice.
- The flexural strength of Clearfil was higher than that of Bisco composites. The microhardness of Clearfil-Dual and Clearfil-Photo were higher compared to other materials.
- The type of polymerization of Bisco core composites did not affect conversion. In areas with limited light access, dual-cured core composites may be recommended instead of self-cured materials.
- Self-cured composites showed greater elution of TEGDMA and BisGMA monomers compared to light-cured and dual-cured materials. DC positively correlated to microhardness and compressive/flexural strength and negatively to BisGMA elution.
- Microhardness may be used as an indicator of elution as a negative correlation was found between elution of both TEGDMA and BisGMA and microhardness.
- Both regional and overall mechanical properties, i.e., microhardness, compressive and flexural strength of core composites positively correlated with monomer-to-polymer conversion.
- Self-cured composites should not be recommended for routine clinical use as their performance was inferior to dual- and light-cured composites.
- Clearfil Dual can be regarded as the most convenient analysed material from the point of mechanical properties and degree of conversion.

## Figures and Tables

**Table 1:** Core composites used in this study.

Material (Code)	Manufacturer	Shade/Lot Number	Type	Composition *
ClearfilTM Core (Clearfil-Self)	Kuraray Noritake Dental Inc., Tokyo, Japan	neutral/041,515	Self-cured	BASE: BisGMA, TEGDMA, Silanated glass filler, Colloidal silica, Accelerators, Pigments, Others CATALYST: BisGMA, HEMA, Benzoyl Peroxide, 10-MDP, Hydrophobic methacrylate, Others
ClearfilTM DC Core Automix (Clearfil-Dual)		white/0090AA	Dual-cured	BisGMA, TEGDMA, Hydrophilic aliphatic dimethacrylate, Hydrophobic aromatic dimethacrylate, Silanated barium glass filler, Silanated colloidal silica, Colloidal silica, Camphorquinone, Aluminum oxide filler, Initiators, Accelerators, Pigments
ClearfilTM Photo Core (Clearfil-Photo)		translucent/2377AA	Light-cured	BisGMA, TEGDMA, Silanated silica filler, Silanated barium glass filler, Camphorquinone, Catalysts, Accelerators
Bisco Core-FloTM (Bisco-Self)	Bisco Inc., Schaumburg, IL, USA	neutral/1,000,005,563	Self-cured	BASE: Ethoxylated-BisGMA, TEGDMA, Glass filler, Silica. CATALYST: BisGMA, TEGDMA, Glass filler.
Bisco Bis-CoreTM (Bisco-Dual)		natural/100,005,781	Dual-cured	BASE: BisGMA, UDMA, Glass filler, Fused silica. CATALYST: BisGMA, TEGDMA, Glass Filler, Fused silica
Bisco Light- CoreTM (Bisco-Photo)		translucent/100,005,781	Light-cured	BisGMA, Ethoxylated-BisGMA, Glass Filler

* Manufacturers data. BisGMA—bisphenol A diglycidyl methacrylate; UDMA—urethane dimethacrylate; TEGDMA—triethyleneglycol dimethacrylate; HEMA—2-hydroxyethyl methacrylate; 10-MDP—10-methacryloyloxydecyl dihydrogenphosphate.

**Table 2:** Differences in the percentage by weight (wt%) of TEGDMA and BisGMA in uncured materials experimentally obtained using high performance liquid chromatography (HPLC) and stated by the manufacturers.

Material	HPLC Data, Mean (SD)		Manufacturers’ Data	
	TEGDMA	BisGMA	TEGDMA	BisGMA
Bisco-Dual base	1.45 (0.35)	9.07 (0.56)	n/a	<15
Bisco-Dual catalyst	4.06 (0.43)	16.46 (1.37)	<20	<20
Bisco-Photo	2.85 (0.21)	10.71 (0.37)	n/a	>5
Bisco-Self base	7.64 (0.29)	10.29 (0.89)	<25	<40
Bisco-Self catalyst	4.70 (0.39)	15.08 (0.48)	<15	<25
Clearfil-Dual base	5.67 (0.45)	12.84 (1.00)	≤2.5	2.5–10
Clearfil-Dual catalyst	2.12 (0.09)	1.52 (0.04)		
Clearfil-Photo	4.02 (0.30)	12.42 (1.86)	<6	<13
Clearfil-Self base	5.34 (0.08)	12.02 (1.15)	5–20	5–25
Clearfil-Self catalyst	3.90 (0.10)	13.37 (0.54)	-	25–50

SD—standard deviation.

## Data Availability

Not applicable.
